# Forming Quality Analysis on the Cold Roll Forming C-channel Steel

**DOI:** 10.3390/ma11101911

**Published:** 2018-10-09

**Authors:** Xiangjun Hui, Xianming Wang

**Affiliations:** 1 College of Mechanical Engineering, Zhejiang Industry Polytechnic College, Shaoxing 312000, China; 2 College of Mechanical Engineering, Zhejiang University of Technology, Hangzhou 310000, China; xwang@zjut.edu.cn

**Keywords:** cold roll forming, C-channel steel, forming passes calculation, bite zone, residual stress, X ray diffractometer

## Abstract

Cold roll forming, as a metal plastic forming process, is still mainly used in industrial production by the trial-and-error method, which wastes a lot of time and materials. In this paper, the C-channel steel is taken as the research object. First, the empirical equations of forming passes are verified and analyzed, then the cold roll forming model of C-channel steel is established, the forming quality of each pass and the stress-strain distribution of the whole sheet metal are analyzed, and the validity of the model is verified by experiments. The residual stresses of the web zone and flange of the finished product were measured. The results show that the empirical formulas are still not universal and the forming quality of the bite zone is poor. It needs to be adjusted by improving the distribution of deformation. The external surface of the C-channel steel is undertensile stress, while the internal surface is undercompressive stress, and the residual stresses of the flange are far greater than those of the web zone. The research provides a reference for the design of the bite zone and the number of forming passes.

## 1. Introduction

Cold roll forming, as an energy-saving and material-saving metal cold forming process, is widely used in construction, automobile, railway, aerospace, and other fields. As a kind of common metal skeleton in construction, the forming quality of C-channel steel is particularly important. However, with the shape of forming products becoming more and more complex, the requirement for forming process is becoming higher and higher. In industry production, technicians also generally design roll flowers by the trial-and-error method, which causes a large number of human and financial losses, and also makes cold roll forming process is still generally considered as art, not science [1,2]. Zeng et al. [3,4] adopted equiradial design method to build a second-order response surface model that represents the relationship between forming angle increment, roll radius, and the maximum edge membrane longitudinal strains. They also established a first-order response surface model between the above parameters and springback. By using the above methods, they optimized the roll profile and also analyzed a twelve passes roll forming process of channel section with an outer edge. Li et al. [5] simulated the U-channel steel cold roll forming process with the dynamic explicit finite element method and obtained the optimized roll diameters by a response surface model. Li et al. [6] analyzed the strip crossing former frame to next one and found that the concentration district of stress exits in transition region and main deforming region below the roll gap central line. Xu and Wang [7] using Prandtl-Reuss flow rule and Mises yield criterion and Updated-Lagrangian increment repeatedly method to study the metal flow ruler from unsteady to steady. Zheng [8] simulated the springback in cold roll forming and found that the mechanical properties of material, sheet thickness, forming angle and roll distance have effects on springback, and the springback law could not be calculated by the general springback formula. Heislitz et al. [9] used PAM-STAMP (a kind of simulation software) to simulate the cold roll forming process. They adopted eight-node solid elements to generate mesh for long sheet and four-node shell elements to simulate roll surface. The results show that the strain distribution after springback can be obtained by PAM-STAMP. Sukmoo et al. [10] used the rigid-plastic finite element method to simulate the forming process, and found that the work hardening coefficient has a very important effect on the forming length, annealed sheet is easier to be formed. By studying the ICF (incremental counter forming) process, Park et al. [11] found that the longitudinal strain distribution and the values could be controlled by the bending angle, which is influenced by the forming parameters. Liu et al. [12] used mathematical models to analyze the distribution of bending angle and longitudinal strain in the deformation process, and the calculated results were in good agreement with the experimental data, which played a role in predicting the longitudinal strain trend in cold roll forming. Jiang et al. [13] found that the number of elements and mesh affects the stability and convergence of the simulation model. Jeong et al. [14] used rigid-plastic finite element method to compare two different roll forming methods, and got a more reasonable roll flower design method. Cai et al. [15] determined the three-dimensional curved surface of sheet metal in continuous roll forming and found that the center line of roll gap has great influence on the forming axis and longitudinal curvature.

Some scholars have studied the cold roll forming process parameters that affect the forming quality. Luo et al. [16] have found that the yield strength, strengthening coefficient, and bending angle increment are sensitive to the springback. Chen et al. [17] analyzed and designed the roll forming machine and found that the springback and longitudinal strain of roll forming 1200 MPa super-high strength steel sheet are more obvious, which need to be overbended and corrected. Bui et al. [18] have studied the material parameters, roll distance, friction coefficient and forming speed in cold roll forming process, and found that forming speed and friction play a minor role to product quality. Bidabadi et al. [19] studied the pre-notched hole strip in cold roll forming and discussed the roll flower, uphill and downhill strategies, roll distance, lubrication, hole distance, hole diameter, flange width, and strip thickness. They found that the forming angle increment was the key factor affecting the hole ovality.

Most of the above researches focused on the reliability of simulation model and the effects of process parameters. Few researches are done on residual stress of cold roll forming. Weng et al. adopted electrical discharge machining technique to cut coupons for residual stress measurement [20]. When compared with the saw-cutting method, it can greatly reduce the external disturbance caused by heating, clamping, and vibration. Li et al. got the residual stress distribution in roll-formed square hollow sections with X-ray diffraction method [21]. Zeng studied the residual stress on welding line and established a prediction model for it [22]. Sánchez Egea et al. used X-ray diffractometer to identify and evaluate the phase transformation induced. Furthermore, considering the thermomechanical effect, they use two different hardening laws to study the residual stress of the wire drawing process with X-ray diffractometer [23,24].

In this paper, the residual stress of C-channel steel are measured by the X-ray diffraction method and predicted by the simulation model that we established. Meanwhile, the whole forming process is analyzed, especially the bite zone. In this paper, the above aspects are studied with the method combined simulation and experiment.

## 2. Materials and Methods

### 2.1. Experiment and Simulation Model Set Up

**Figure 1:**
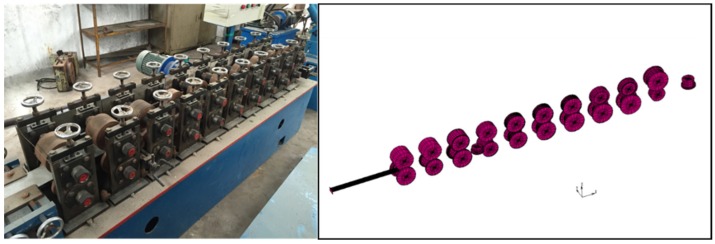
The finite element simulation and the mill of cold roll forming.

**Figure 2:**
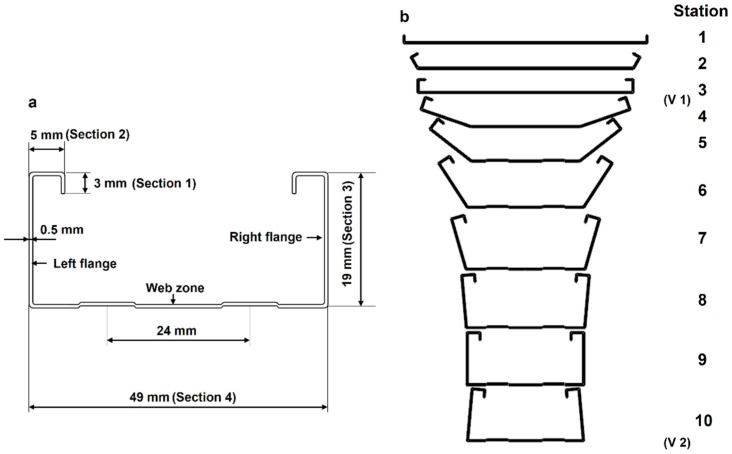
(**a**) The basic dimensions of C-channel steel; (**b**) Flower pattern.

The cold roll forming machine that was used in this experiment is shown in Figure 1. It consists of ten passes vertical rolls and two passes horizontal rolls. The simulation model is established in MSC.Marc. 2014 (MSC.Marc. is a full-featured advanced nonlinear finite element software). The material used in the simulation is Q235. The constitutive equation is shown in Equation (1) [4], where $\bar{\sigma }$ is the plastic flow stress and $\bar{\varepsilon }$ is the strain. The chemical composition and the mechanical properties are shown in Table 1 and Table 2. The cross section dimensions of the C-channel steel and the ten passes roll flowers are shown in Figure 2, in which V1 and V2 are two passes horizontal rolls for straightening.

(1) $$\bar{\sigma }=617.2{\left(0.001292+\bar{\varepsilon }\right)}^{0.143}$$

The simulation parameters that were established in MSC.Marc. are as follows: the Young’s modulus 210 GPa, Poisson’s ratio 0.3, the dimensions of the strip model are: length 500 mm, width 47.5 mm, and thickness 0.5 mm. The coulomb friction model has been used in the simulation model. The friction coefficient was assumed as 0.2 according to the Zeng et al. [4], and the forming pass distance is 210 mm.

### 2.2. Residual Stress Measurement

**Figure 3:**
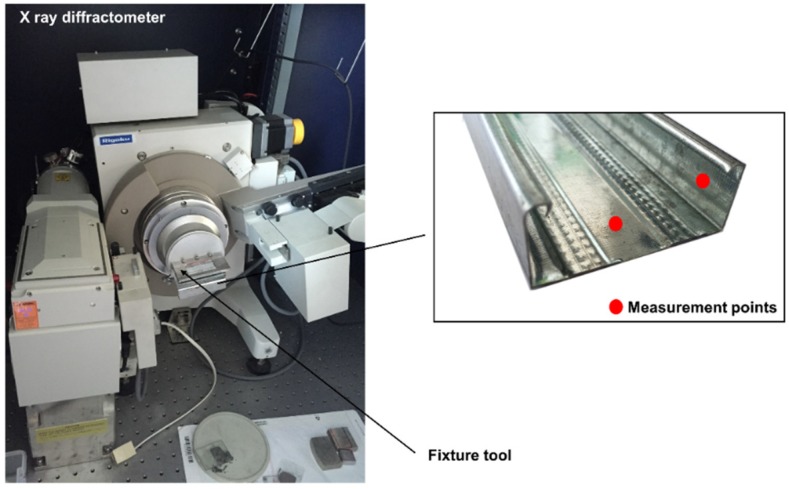
Residual measurement device and measurement locations on the specimen.

**Figure 4:**
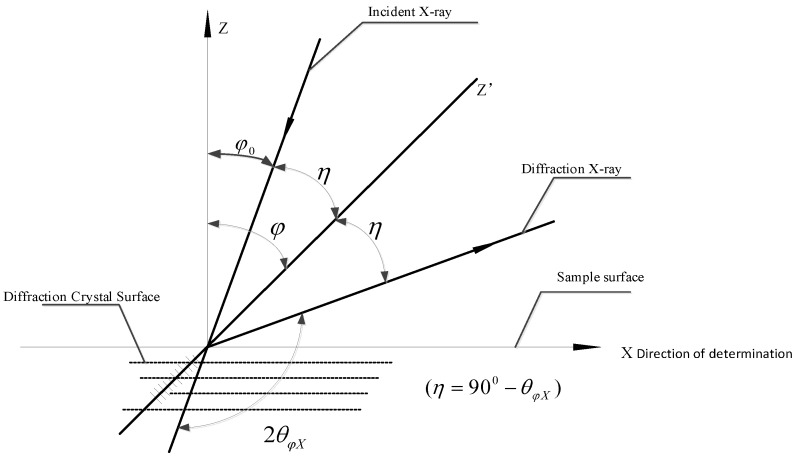
Schematic diagram of residual stress measurement.

In this paper, residual stress is adopted as an index to test the forming quality of C-channel steel. Residual stresses are mainly divided into macro residual stress, micro residual stress, and the third kind of internal stress. The macro residual stress is measured in this paper. The residual stress is measured by X-ray diffractometer see Figure 3. Rigaku D/max-2500PC (Ningbo, China) is the X-ray diffraction device that we used to measure the residual stress. The power of the X-ray generator is 18 Kw. The measurement range of 2*θ* is −10°–158° and the parameters are shown in Table 3. Considering the work of Sánchez Egea et al. [23], a Cu radiation was used. The residual stress was measured with the angle from 81.5°–84°with step size of 0.1° and measuring time of 5 s per step. According to GB/T 7704-2008 [25], the measuring principle is shown in Figure 4.

The residual stress is measured according to the variation of the crystal plane spacing. For Q235 steel, the deformation of the sheet under external force will cause the distortion of crystal lattice in three-dimensional direction, which will cause the peak position shift and the change of half-peak width. From the Bragg equation 2dsinθ=nλ, where *d* is the crystal plane spacing, 2*θ* is the diffraction angle, *λ* is the wavelength and *n* is the order of reflection, the strain and stress can be deduced.

While the residual stress of the selected direction can be calculated by the following equation:

(2) $$\sigma_{X}=K\cdot M$$

(3) $$K=-\frac{E}{2(1+\nu )}\cot\theta_{0}\frac{\pi }{180{}^{\circ}}$$

where $\sigma_{X}$ is the residual stress in X direction, while *K* is a stress constant, *E* represents the Young’s modulus, *v* is the Poisson ratio, $\theta_{0}$ denotes the bragg angle at unstressed state, and *M* is the slope of line $2\theta_{\varphi X}-\sin^{2}\varphi$. Where φ represents the azimuth angle of diffraction crystal plane, and $2\theta_{\varphi X}$ is the diffraction angle for selected φ.

The workpiece in Figure 4 is a part of C-channel steel. The measuring position are web zone and flange while the bending zone is difficult to measure because of its small size.

## 3. Forming Passes Design and Analysis

There have several calculation methods for the number of forming passes:

(4) $$n=\frac{\sqrt{2a^{2}}}{d\tan\alpha_{1}},$$

While *α*_1_ is the estimated angle (range 1°~1.5°), *a* is the bending edge length, and *d* represents the roll distance.

(5) $$n=\left[0.237h^{0.8}+\frac{0.834}{t^{0.87}}+\frac{\alpha_{2}}{90}\right]{\left[\frac{Y^{2.1}}{0.003U}\right]}^{0.15}s\left(1+0.5z\right)+e+f+5zs,$$

where *h* is the maximum height of cross section, *t* is the thickness of material, *α*_2_ represents the total forming angle, *Y* is the yield stress, *U* is the tensile strength, and *z* represents the pre-punching/punching and continuous coefficient of sheet metal, sis the shape coefficient, *e* is the additional number of forming passes, and *f* is the tolerance coefficient.

(6) $$\phi =Fn_{1}t,$$

For *φ* is the shape factor function, *F* is the sum of the lengths of left and right vertical edges, *n*_1_ represents the total bending angle of the section, and *t* is the thickness of material.

(7) n=(H/L)cotθ,

While *H* is the bending height, *L* is the frame distance, and *θ* is the forming angle.

Equation (4) is the original equation proposed by Halmos [2], which is the earliest equation used for calculating the forming passes. It is calculated from a simple linear model. Its principle is to calculate the total distance from the first pass to the end pass of cold roll forming, and then determine the roll distance to get the required number of forming passes. However, this equation only provides a rough evaluation without considering the key factors, so the result is not very reliable. Halmos [2] proposed an empirical Equation (5), but it has not been completely verified. Hiroshi et al. [1] summarized Equation (6) according to the data graph that was given by Japan Iron and Steel Association with the shape factor method, and calculated the forming passes with the specific data graph. Zhao [26] proposed Equation (7) to determine forming passes by bending height, frame distance, and forming angle. In this paper, the cold bending machine used in the research is used to verify the number of forming passes, the results are shown in Table 4.

## 4. Results and Discussion

### 4.1. Simulation Analysis of the Forming Process on the C-Channel Steel

Cold roll forming is a process of metal slow deformation due to its special forming length. The front part of the sheet is always formed faster than that of the back part. On the whole, the sheet metal is in a state of gradually twisting and bending. The stress and strain distribution analysis of the sheet metal is helpful to understand the dangerous area and the whole forming process. The dangerous area is a region where critical levels of strains are reached, especially at the bending zone of the C-channel steel. In this area, the strain value is larger than the other zones, and the materials in this area flow to the adjacent areas. So, the thickness reduction in this area is large, which may cause cracks. It is convenient for the overall improvement of the roll flowers design.

**Figure 5:**
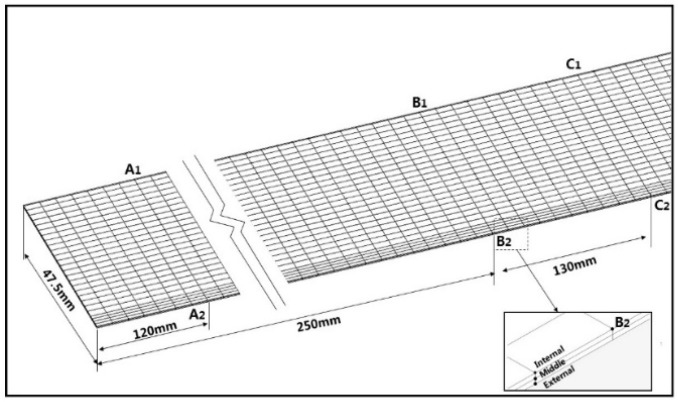
Node positions of the sheet metal model.

**Figure 6:**
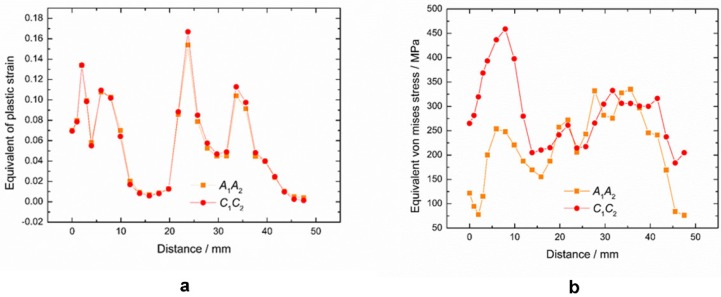
(**a**) Equivalent strain distribution and strain comparison; (**b**) Equivalent stress distribution and stress comparison between front part to behind part of the strip.

The sheet metal model is marked, as shown in Figure 5, and the front part nodes A_1_, A_2_, and the back part nodes C_1_, C_2_ are taken as the measuring nodes. As we can see from B_2_, the sheet mesh in thickness direction is divided into three layers and the local grid refinement is used for a better forming effect of Section 1. Figure 6a,b show that the maximum peak strain (i.e., the bending area between the web zone and the flange) in the front part of the sheet is smaller than that in the back part, and the stress distribution in the front part is also smaller than that in the back part. The main reason is the work hardening in cold forming of the sheet metal. The sheet has undergone cyclic loading and unloading. Because of the deformation of the front part, the deformation of the back part tends to the shape of the roll before it enters the roll, and the deformation of the deformed back part occurs when it contacts the roll. At this time, the deformation of the sheet metal will be more difficult to form due to the material work hardening, so the required stress is also greater than that of the front part (see Figure 6b). As a result, the forming of the back part is more thorough, the thinning is larger, and the peak strain is relatively larger.

**Figure 7:**
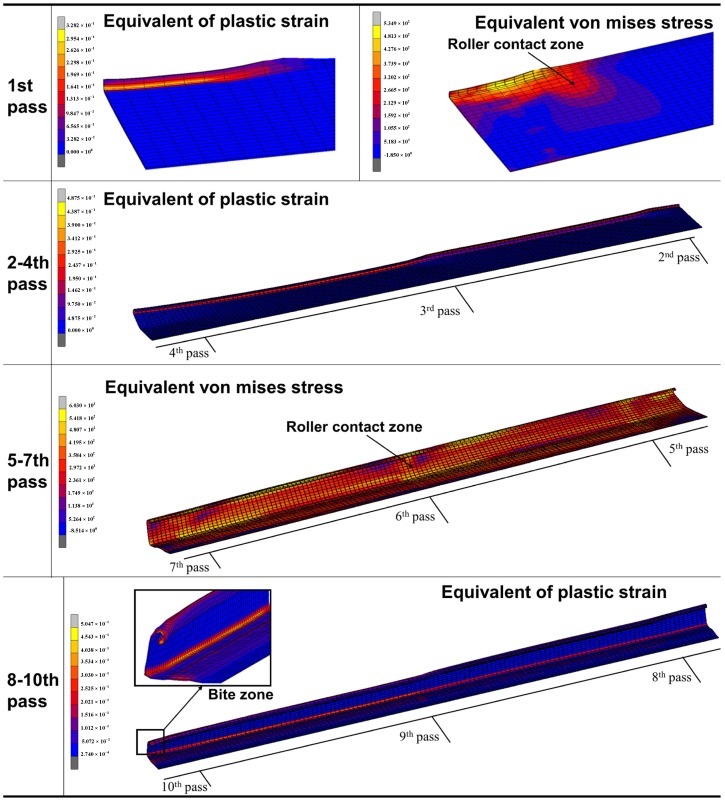
The forming quality of the bite segment.

However, forming quality at the beginning of the sheet (the bite zone) is poor. As shown in the Bite zone in Figure 7, the crimp of the bite zone is very obvious and the bending angle is too large to meet the forming dimension requirement. This is mainly due to the fact that the bite zone is the initial part of the forming sheet and it directly faces the straight roll shape during the forming process. The back part has already deformed to the shape of the roll due to the deformation of the front part. From the strain and stress distribution of the 1st pass in Figure 7 can be seen, in the back area of the roll contact zone, there is strain and stress, which has caused the uncontact-roll area deformation, and it makes the forming process has transitional stage, and the forming quality is relatively good. From the strain distribution of 2–4th passes, it can be seen that the main forming parts of these passes are the Section 1 in Figure 2. It can be seen that the high strain zone is located at the bending edge of Section 1, where the forming dimension error is large. The stress distribution of 5–7th passes shows that the stress at the roll contact zone is bigger and the stress on both sides is radially decreased. The stress distribution in lateral of the sheet is based on the roll pass distance. The larger stress is focused on the roll contact zone, while stress of the sheet between two passes is smaller because there has no direct roll actions. The stress is larger at the upper and lower bending zone of the flange. The sheet stress in longitudinal direction is mainly concentrated in the bending zone that is directly in contact with the roll, and the stress on the center of the web zone and the flange is relatively small. The strain distribution of 8–10th passes shows that the bending zone of the flange is a high strain concentration area, where the sheet thinning is the largest, and it is a dangerous area for the design of the C-channel steel.

### 4.2. Residual Stress Analysis on the C-Channel Steel

**Figure 8:**
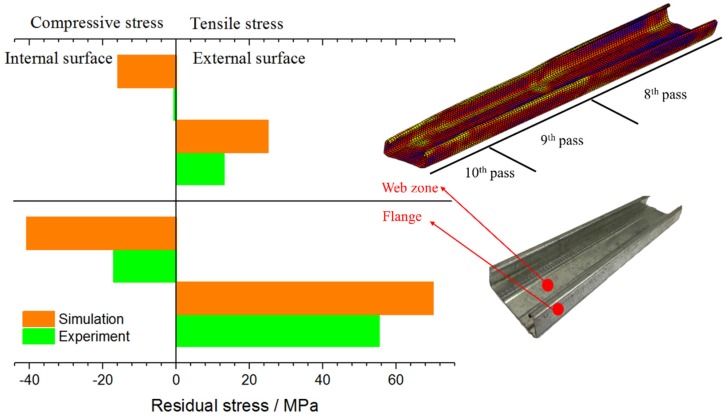
Comparison of the numerical and the experimental residual stress.

The measurement of residual stress mainly concentrates on the internal and external surfaces of web zone and flange. Figure 8 shows the experimental and numerical results of the residual stress on the internal and external surfaces corresponding to the measurement points shown in Figure 8. The simulation results have a certain error when compared with the experimental results. It can be seen that the residual stress in the web zone is significantly less than the flange, and the residual stress on the external surface is slightly larger than the internal surface. The comparison between the simulation model and the experimental samples can further prove the reliability of the simulation model, so the model can be used to predict the residual stress. The reasons for the errors are as follows, for simulation errors: (i) The mesh size of the sheet, especially at the bending zone. More layers and smaller size of the elements are needed to improve the simulation results. (ii) The assumed friction coefficient might not proper. A more accurate friction coefficient is needed. For measurement errors: (i) Q235 is used as material parameters in the simulation model, but a layer of zinc is deposited on the surface of the material, which is not reflected in the simulation model. The zinc coating may interfere with the results of X-ray diffractometer measurement; (ii) there has a certain degree of residual stress release of the measurement sample that is used in experiment because of the cutting. To improve the measurement results, the sample can be measured by mechanical measurement method, such as the hole-drilling method. However, it is a kind of destructive measurement method.

## 5. Conclusions

(1) The forming quality of the bite zone is the worst in the whole cold roll forming process. The analysis of the layered distribution of sheet shows that the external layer is mainly subject to tensile stress, while the internal layer is mainly subject to compressive stress, and the internal layer residual stress is slightly larger than the external layer. It offers a reference for the forming of anisotropy material and the material with different coatings at internal and external surfaces.
(2) The equations for calculating forming passes that are proposed by many scholars are based on the materials and shapes of the products they studied and lack of universality. They can only predict the number of forming passes roughly and verified by experience.
(3) In order to save costs, one forming pass can be used to shaping the short edge of the target product (such as Section 1 in Figure 2a 3 mm short edge in this paper). However, the forming shape and dimension accuracy are not very good. This design method is suitable for those products that do not require high quality at the short edges. For the high quality and accuracy dimension products, more forming passes are needed for the short edge forming.
(4) For long edge forming of the target product (such as Section 3 in Figure 2a 19 mm long edge), several forming passes (seven passes are used in this paper) are needed to avoid the dangerous area and keep the dimension accuracy. An over bending pass is needed to compensate the material springback, especially in the long edge forming.
(5) The forming process in this paper offers a reference for the forming pass design of U-channel and V-channel steel.
(6) Residual stress measurement of C-channel steel shows that the external surface of C-channel steel is mainly tensile stress, the internal surface is mainly compressive stress, and the residual stress of flange is far greater than that of web zone, which hardly deforms during cold roll forming and residual stress is very small.

For future work, we want to find the relationship between the residual stress prediction values and the dangerous level of the dangerous areas, and use the simulation model and measurement equipment to predict and check whether the products meet the requirements. For this purpose, conclusion (6) provides a theoretical basis.

## Figures and Tables

**Table 1:** Chemical composition of Q235 steel (%).

C	S	Si	Mn	P
0.150	0.035	0.300	0.350	0.035

**Table 2:** Mechanical properties of Q235 steel.

Yield Strength (MPa)	Tensile Strength (MPa)	Elongation (%)
235	423	26

**Table 3:** Measurement parameters of residual stress.

Parameter	Value
Bragg crystal angle	82.33°
Crystal surface	211
Wavelength	1.54 angstrom
Light seams	1 mm

**Table 4:** Comparison of calculation methods on forming passes.

Calculation Method	Number of Forming Passes
Equation (4)	10
Equation (5)	14
Equation (6)	9
Equation (7)	8

## References

[1] Hiroshi O., Liu J.Y. (2008). Cold Forming Technology.

[2] Halmos G.T. (2009). Roll Forming Handbook.

[3] Zeng G., Li S.H., Yu Z.Q., Lai X.M. (2009). Optimization design of roll profiles for cold roll forming based on response surface method. Mater. Des..

[4] Zeng G., Lai X.M., Yu Z.Q., Guo Y.J. (2007). The simulation of roll forming with multiple passes. J. Shanghai Jiaotong Univ..

[5] Li D.Y., Jiang J.M., Peng Y.H., Yin J.L. (2007). Study on roll forming process simulation and roll diameters optimization. J. Syst. Simul..

[6] Li B., Zhang S.H., Hu L. (2004). Numerical simulation on cold roll forming process of profiled strip. Iron Steel.

[7] Xu S.C., Wang X.J. (1999). Analysis of displacement and velocity field in channel steel roll forming process using elastic-plastic large-deformations finite element method. J. Plast. Eng..

[8] Zheng J.X. (2006). Finite Element Method Simulation and Springback Research on Cold Roll Forming. Master’s Thesis.

[9] Heislitz F., Livatyali H., Ahmetoglu M.A., Kinzel G.L., Altan T. (1996). Simulation of roll forming process with the 3-D FEM code PAM-STAMP. J. Mater. Process. Technol..

[10] Sukmoo H., Seungyoon L., Naksoo K. (2001). A parametric study on forming length in roll forming. J. Mater. Process. Technol..

[11] Park J.C., Yang D.Y., Cha M.H., Kim D.G., Nam J.B. (2014). Investigation of a new incremental counter forming in flexible roll forming to manufacture accurate profiles with variable cross-sections. Int. J. Mach. Tool. Manuf..

[12] Liu C.F., Zhou W.L., Fu X.S., Chen G.Q. (2015). A new mathematical model for determining the longitudinal strain in cold roll forming process. Int. J. Adv. Manuf. Technol..

[13] Jiang Z.Y., Tieu A.K. (2011). A simulation of three-dimensional metal rolling processes by rigid-plastic finite element method. J. Mater. Process. Technol..

[14] Jeong S.H., Lee S.H., Kim G.H., Seo H.J., Kim T.H. (2008). Computer simulation of U-channel for under-rail roll forming using rigid-plastic finite element methods. J. Mater. Process. Technol..

[15] Cai Z.Y., Li L.L., Wang M., Li M.Z. (2014). Process design and longitudinal deformation prediction in continuous sheet metal roll forming for three-dimensional surface. Int. J. Precis. Eng. Manuf..

[16] Luo X.L., Yu Z.Q., Li S.H., Zeng G. (2008). Finite element analysis of the springback in high strength steel roll forming. J. Plast. Eng..

[17] Chen Z.Y., Wang H.B., Yan Y., Jia F.H. (2014). Design and analysis of roll-forming machine based on ABAQUS equivalent simplified model. Forg. Stam. Technol..

[18] Bui Q.V., Ponthot J.P. (2008). Numerical simulation of cold roll-forming processes. J. Mater. Process. Technol..

[19] Bidabadi B.S., Naeini H.M., Tafti R.A., Mazdak S. (2015). Experimental investigation of the ovality of holes on pre-notched channel products in the cold roll forming process. J. Mater. Process. Technol..

[20] Weng C.C., Pekoz T. (1990). Residual stress in cold-formed steel members. J. Struct. Eng..

[21] Li S.H., Zeng G., Ma Y.F., Guo Y.J., Lai X.M. (2009). Residual stresses in roll-formed square hollow sections. Thin Wall Struct..

[22] Zeng G. (2009). Simulation and Experimental Study on Residual Stresses for Multi-Stand Roll-Formed Sections. Ph.D. Thesis.

[23] Sánchez Egea A.J., González Rojas H.A., Celentano D.J., Peiró J.J. (2016). Mechanical and metallurgical changes on 308L wires drawn by electropulses. Mater. Des..

[24] Sánchez Egea A.J., González Rojas H.A., Celentano D.J., Peiró J.J., Cao J. (2017). Thermomechanical analysis of an electrically-assisted wire drawing process. J. Manuf. Sci. Eng..

[25] (2008). Non-Destructive Testing-Practice for Residual Stress Measurement by X-Ray.

[26] Zhao Y.H. (2003). Design way to the forming roller of cold-forming profiled bar. Shanxi Mach..

